# Sexual dysfunctions in patients with diabetes: a study from Iran

**DOI:** 10.1186/1477-7827-8-50

**Published:** 2010-05-18

**Authors:** Marzieh Ziaei-Rad, Mariam Vahdaninia, Ali Montazeri

**Affiliations:** 1 Faculty of Nursing and Midwifery, Islamic Azad University, Khorasgan Branch, Isfahan, Iran; 2 Department of Social Medicine, Iranian Institute for Health Sciences Research, ACECR, Tehran, Iran; 3 Department of Mental Health, Iranian Institute for Health Sciences Research, ACECR, Tehran, Iran

## Abstract

**Background:**

Diabetes mellitus is a chronic disease that causes short and long-term complications. This study aimed to investigate the prevalence of sexual dysfunctions (SD) among diabetic patients in Iran and to examine whether glycemic control has a role in SD.

**Methods:**

A consecutive sample of diabetic women and men who were registered in the Isfahan Endocrine and Metabolism Center, Iran were studied. Sexual dysfunction was evaluated using the Female Sexual Function Index (FSFI) in women and the International Index of Erectile Function (IIEF) in men. In addition the level of glycosylated hemoglobin was assessed to classify the diabetes status in patients.

**Results:**

In all 200 patients (100 male and 100 female) were entered into the study. The mean age of patients was 48.6 (SD = 7.3) years and most had type 2 diabetes (91.0%). The results showed that sexual dysfunctions were widespread in both gender and 165 (82.5%) patients reported that experienced at least one sexual dysfunction. There were significant associations between sexual dysfunctions and gender and type of diabetes (P = 0.04). Women and patients with type 1 diabetes had higher rates of SD. No major differences were found between SD and age, diabetes status, duration of diabetes and hypertension. In addition, glycemic control did not show a significant association with SD in both genders.

**Conclusion:**

The findings of this study showed that SD prevalence was high in diabetic patients of both genders and the glycemic control did not correlate with the frequency of SD in the study population. It is recommended that SD should be addressed more precisely in health care practice in Iran.

## Background

Diabetes currently affects 246 million people in world and is expected to affect 380 million by 2025. In addition by 2025 the largest increases in diabetes prevalence will take place in developing countries [1]. Hyperglycemia in diabetic patients can cause short and long term complications. The long-term problems occur 5-10 years after diagnosis in both types of diabetes and could be prevented or postponed by precise controlling of blood sugar (BS) level. The amount of BS can be monitored daily either by patient or clinician while in long-term method glycosylated hemoglobin (GH) is checked [2]. GH is a blood test that reflects average blood glucose levels over a period of approximately two to three months after its raise. The longer the amount of glucose in the blood, the more binding of glucose to hemoglobin is expected [2]. Therefore the range of GH is an objective measurement for controlling diabetes in patients with the disease. There are more supplementary recommended standards of medical care in patients with diabetes: measurement of blood pressure at every routine diabetes visits, annual measurement of fasting lipid profile, screening for risk factors of: coronary heart disease, neuropathy, retinopathy, nephropathy and foot care. In addition, it has been advised that people with diabetes should receive individualized medical nutrition therapy and the usage of fat and carbohydrate must be limited [3].

Diabetes' complications can affect nervous system including a group of ailments that involve peripheral, autonomous and spinal cord nerves. Autonomic nervous system imperfections accounts for a vast range of these disorders that would engage all body systems including cardiovascular, gastrointestinal, urinary, adrenalin glands and reproductive system [2]. Their prevalence is associated with age, weight, disease duration, GH level, cholesterol level, hypertension and smoking. In all diabetes could affect patients' life in several ways. For instance since patients with diabetes should receive individualized medical nutrition thus one might expect that patients change their regular nutrition habits. In addition the stigma associated with a chronic disease such as diabetes might have effect on patients' social life and perhaps cause social isolation. It is well recognized that social isolation is an important risk factor for depression [4].

Sexual dysfunction (SD) as a diabetes-related difficulty is common among both male and female patients [5,6]. Despite over 70 years of research, sexuality of females with diabetes mellitus remains a controversial issue. Despite over 70 years of research sexuality of females with diabetes mellitus remains a controversial issue. There is a debate that type of diabetes has an impact on the emergence of SD in women with diabetes. Research indicates that women with type 1 diabetes are mostly affected by SD compared to type 2 diabetics and healthy women [7]. Overall studies suggest women with diabetes report more sexual dysfunctions compared to their counterparts and increased diabetes related complications can intensify the SD problems in patients [8]. Sexual problems in women with diabetes mostly include sexual desire, sexual satisfaction, orgasmic disorder, arousal disorder and lubrication [9,10]. It seems that somatic sensory system is affected by diabetes and intruitos vagina, labium minora and clitoris are the most deteriorated genital sites in diabetic women. Even though manifesting of sexual difficulties is not always expected and medication can improve blood flow in clitoris [11,12]. It is argued that neuropathy, vascular impairment, and psychological complaints are recognized factors in the pathogenesis of female sexual dysfunction in diabetic women [13]. In addition endocrine changes may cause diminished vaginal lubrication in women who suffer from diabetes; however it has rarely been documented as a sexual problem in female sufferers [14]. In men with diabetes erectile dysfunction is more frequent than other men of the same age [15]. Diabetes causes damage to nerves throughout the body including penis and erectile dysfunction generates as common diabetes' complication [16]. A number of men with autonomic neuropathy can experience normal erectile function and orgasm but do not ejaculate normally that could be verified through urine analysis [2]. Research indicates although erectile dysfunction is widespread among men with diabetes; the condition is often remains undiagnosed and demands appropriate assessment and initiation of proper treatment [17]. A study by Zdravko supported the hypothesis that in the complex pathogenesis of diabetic erectile dysfunction (ED), diabetic neuropathy is the major pathogenic factor [18]. Other research findings are indicative that the etiology of ED is a multifactor disorder and in the management of diabetic ED, a holistic approach should be applied [19].

This study aimed to assess the differences between controlled and uncontrolled diabetic cases of both gender in Isfahan, Iran.

## Methods

### Participants and data collection

A cross-sectional study was conducted among 200 diabetic patients of both genders who were registered in Isfahan Endocrine and Metabolism Center. The sample was selected in a consecutive procedure from November 2002 to May 2003. The sample size was estimated on the basis of a single proportion design. We assumed that at best 50% of the patients would have controlled diabetes status. Thus, a study with a sample of 206 diabetic patients would have 80% power to detect a difference of 5% (45-55%) at the 0.05 significance level. The sample size actually obtained for this study was 200 (100 men and 100 women with diabetes).

The subjects were categorized into two groups: female and male and based on their level of GH were set into controlled (GH less than 10%) and uncontrolled (GH more than or equal to 10%) clusters [20]. The subjects were matched with regard to their age, type and duration of diabetes. The inclusion criteria were identified as: registered for a year or longer (these patients had received all their two to three recent HbA1c tests in a year that let the researcher to use the mean of GH test in the study), history of diabetes for ten years or longer [21], existing of at least two GH analyses in their case records, being married and aged 30 and over (Most of the registered patients in Isfahan Endocrine and Metabolism Center are with type 2 diabetes and the disease is most prevalent among 35 years and over. Therefore the age range ≥30 was defined as inclusion criteria).

Exclusion criteria included: record of mastectomy, total hysterectomy, prostate and pelvic surgeries, benign prostate hypertrophy (BPH), prostate cancer, Peyronie's disease, pregnancy, polygamy, patients who by himself or their spouses were rising up in single parent families [22], existence of sexual disorders in patients' spouse, anemia and presence of sexual disorders before getting diabetes. However, we did not control the presence of depressive symptoms and type of medication.

### Measures

1. Sexual dysfunction: sexual dysfunctions were measured in women and men using standard questionnaires. The Female Sexual Function Index (FSFI) is a known instrument that assesses sexual function in women with six domains [23]: desire, arousal, lubrication, orgasm, satisfaction and pain during sexual intercourse. The shorter form (5 items) of the International Index of Erectile Function (IIEF) [24] was used to measure SD in men. It evaluates sexual function in five areas: erectile function, orgasmic function, sexual desire, intercourse satisfaction and overall satisfaction. Due to cultural issues we did not ask about 'intercourse satisfaction' from male patients. Each item is rated on a 5-point scale. For the purpose of analysis, relative to responses patients were divided into two groups: with sexual problems, and without SD. In women, the FSFI score <17 and in men the IIEF score <14 were the criteria for accepting the presence of sexual dysfunction.

2. Clinical measures: we used available data from Isfahan Metabolism and Endocrine Centre where all patients received routine clinical examination that included recording of: duration of diabetes, type of diabetes, body mass index (BMI), fasting blood sugar, previous GH tests, blood pressure, cholesterol and triglycerides levels and diabetes-related complications. The micro-vascular complications included recording of retinopathy, nephropathy, and neuropathy (peripheral, autonomic), whereas macro-vascular complications included recording of atherosclerosis, heart disease and stroke. All hypertensive cases were being treated with antihypertensive regimes. In addition, morning urine analysis was carried out in men as a test for evaluating retrograde ejaculation disorder [2]. It was expected that lots of active sperms would be found in the morning urine in men with the disorder.

3. Demographic data: this included recording of patients' age, occupation and socio-economic status. Socioeconomic position was estimated using three indicators: income, home ownership and family size. Patients were categorized into two groups: those with good conditions (good) and those with intermediate or poor conditions (less than good).

### Statistical analysis

Data were analysed using SPSS 15 in a descriptive and analytical fashion. To investigate the associations of demographic and clinical characteristics of the patients with SD the χ^2 ^test was used.

### Ethical considerations

The ethics committee of Isfahan Endocrine and Metabolism Center approved the study. All patients were entered into the study after an informed verbal consent. Diabetic women and men were informed by female and male interviewers separately and were assured regarding the confidentiality of the information.

## Results

In all 200 cases (100 men and 100 women) were studied. The mean age of patients was 48.6 (SD = 7.3). The mean age of men was 50.9 while it was 46.3 for women. Most patients had type 2 diabetes (91.0%). Demographic and clinical characteristics of the study sample are shown in Table 1.

**Table 1:** Demographic and clinical characteristics of study sample (n = 200)

	Total (n = 200)	Men (n = 100)	Women (n = 100)
	No (%)	No (%)	No (%)
**Age group (years)**			
30-39	27(100.0)	12(44.5)	15(55.5)
40-49	84(100.0)	28(33.3)	56(66.7)
50-59	89(100.0)	60(67.4)	29(32.6)
Mean (SD)	48.6(7.3)	50.9(8.3)	46.3(5.2)
**Occupation**			
Employed	107(100.0)	100(93.4)	7(6.6)
Housewife	93(100.0)	0.00	93(100.0)
**Economic status**			
Good	41(100.0)	20(48.8)	21(51.2)
Less than good	159(100.0)	80(50.3)	79(49.7)
**Duration of diabetes (years)**			
10-14	160(100.0)	75(46.8)	85(53.2)
15-19	31(100.0)	21(67.7)	10(32.3)
20-25	9(100.0)	4(44.4)	5(55.6)
**Type of diabetes**			
Type1	18(100.0)	9(50.0)	9(50.0)
Type2	182(100.0)	91(50.0)	91(50.0)
**Diabetes status**			
Controlled	100(100.0)	50(50.0)	50(50.0)
Uncontrolled	100(100.0)	50(50.0)	50(50.0)
**Hypertension**			
Yes	21(100.0)	13(61.9)	8(38.1)
No	179(100.0)	87(48.6)	92(51.4)
**Triglyceride**			
≤200	127(100.0)	76(59.8)	51(40.2)
201≥	73(100.0)	24(32.9)	49(67.1)
**Cholesterol**			
≤200	53(100.0)	35(66.0)	18(44.0)
201≥	147(100.0)	65(44.2)	82(55.8)
**FBS**			
60-130	63(100.0)	34(53.9)	29(46.1)
131≥	137(100.0)	66(48.2)	71(51.8)
**BMI**			
≤20	123(100.0)	50(40.6)	73(59.4)
21-25	58(100.0)	31(53.4)	27(46.6)
26≥	19(100.0)	12(63.1)	7(36.9)

Table 2 presents the relationship between SD and demographic and clinical characteristics of the study sample. The results showed that there was a significant association between presence of SD and gender (P = 0.04) where women were more affected by SD compared to men (88 versus 77%). Higher age groups experienced elevated rate of SD, although there was no significant difference among different age groups (P = 0.13). Uncontrolled cases and patients with hypertension reported higher incidence of SD. In addition diabetes duration did not show a significant association with experience of SD. However all patients with diabetes type 1 had SD and a significant association was found between SD and type of diabetes (P = 0.04).

**Table 2:** Sexual dysfunction (SD) by gender, age, diabetes status, duration of diabetes, type of diabetes and hypertension

	With SD (n = 165)	Without SD (n = 35)	P
	No (%)	No (%)	
**Gender (n = 200)**			0.04
Women (n = 100)	88 (88.0)	12 (12.0)	
Men (n = 100)	77 (77.0)	23 (23.0)	
**Age groups (years)**			0.13
30-39	15(68.2)	7(31.8)	
40-49	74(82.2)	16(17.8)	
50-59	76(86.4)	12(13.6)	
**Diabetes status**			0.35
Controlled	80(80.0)	20(20.0)	
Uncontrolled	85(85.0)	15(15.0)	
**Duration of diabetes (years)**			0.64
10-14	130(81.2)	30(18.8)	
15-19	27(87.1)	4(12.9)	
20-25	8(88.8)	1(11.2)	
**Hypertension**			0.31
Yes	19(90.5)	2(9.5)	
No	146(81.6)	33(18.4)	
**Type of diabetes**			0.04*
Type 1	18 (100.0)	0.00	
Type 2	147(80.8)	35(19.2)	

*Fisher's exact test

The frequency of SD items in men with diabetes for each group are shown in Table 3. Men in controlled and uncontrolled groups did not show significant differences. However, except for erectile function where the frequency was the same between both groups, in other SD items cases with uncontrolled diabetes had higher dysfunctions.

**Table 3:** Frequency of Sexual Dysfunction (SD) scales in men with diabetes

	Total (n = 100)	Controlled (n = 50)	Uncontrolled (n = 50)	OR* (95% CI**)	**P **(χ^2^**)**
	No (%)	No (%)	No (%)		
**Erectile dysfunction**				1.00 (0.28-3.53)	1.00 (0.00)
Yes	14(100.0)	7(50.0)	7(50.0)		
No	86(100.0)	43(50.0)	43(50.0)		
**Orgasmic dysfunction**				0.78 (0.16-3.66)	0.72 (0.12)
Yes	9(100.0)	4(44.4)	5(55.6)		
No	91(100.0)	46(50.5)	45(49.5)		
**Sexual desire**				0.56 (0.33-1.35)	0.15 (2.03)
Yes	59(100.0)	26(44.0)	33(56.0)		
No	41(100.0)	24(58.5)	17(41.5)		
**Overall satisfaction**				0.49 (0.20-1.23)	0.09 (2.78)
Yes	64(100.0)	28(43.7)	36(56.3)		
No	36(100.0)	22(61.1)	14(38.9)		
**Retrograde ejaculation**				0.62 (0.18-2.13)	0.40 (0.71)
Yes	15(100.0)	6(40.0)	9(60.0)		
No	85(100.0)	44(51.8)	41(48.2)		
**Decreased seminal fluid**				0.62 (0.18-2.13)	0.40 (0.71)
Yes	15(100.0)	6(40.0)	9(60.0)		
No	85(100.0)	44(51.8)	41(48.2)		

*Odds ratio

**Confidence interval

Table 4 shows the frequency of SD in women with diabetes for each group. The findings signified that uncontrolled patients showed higher dysfunctions in all items except for satisfaction disorders. Furthermore no significant associations were found in SD between controlled and uncontrolled female patients.

**Table 4:** Frequency of Sexual Dysfunction (SD) scales in women with diabetes

	Total (n = 100)	Controlled (n = 50)	Uncontrolled (n = 50)	OR* (95% CI**)	**P **(χ^2^**)**
	No (%)	No (%)	No (%)		
**Desire disorders**				0.84 (0.34-2.08)	0.67 (0.18)
Yes	66(100.0)	32(48.5)	34(51.5)		
No	34(100.0)	18(52.9)	16(47.1)		
**Arousal disorders**				0.47 (0.09-2.29)	0.29 (1.10)
Yes	9(100.0)	3(33.3)	6(66.7)		
No	91(100.0)	47(51.6)	44(48.4)		
**Lubrication disorders**				0.81 (0.30-2.18)	0.64 (0.21)
Yes	26(100.0)	12(46.2)	14(53.8)		
No	74(100.0)	38(51.3)	36(48.7)		
**Orgasmic disorders**				0.26 (0.03-1.46)	0.08 (3.05)
Yes	9(100.0)	2(22.3)	7(77.7)		
No	91(100.0)	48(52.7)	43(47.3)		
**Satisfaction disorders**				1.61 (0.47-5.00)	0.40 (0.71)
Yes	85(100.0)	44(51.8)	41(48.2)		
No	15(100.0)	6(40.0)	9(60.0)		
**Pain disorders**				0.52 (0.20-1.33)	0.13 (2.29)
Yes	31(100.0)	12(38.7)	19(61.3)		
No	69(100.0)	38(55.1)	31(44.9)		

*Odds ratio

**Confidence interval

## Discussion

This study examined SD using standard measures among 200 diabetic women and men. Most cases (91.0%) had diabetes type 2 and findings indicated that patients in both genders were greatly affected by SD problems. However the incidence of SD among women was higher than men. A study on diabetic female and male cases found that the prevalence of sexual dysfunction in diabetic men approached 50% whereas in diabetic women it was slightly lower [13]. Overall high rate of SD in patients with diabetes are reported in different studies. A study from Iran showed that diabetes significantly impairs the sexual performance of women with diabetes [25]. A study by Doruk demonstrated that female sexual dysfunctions involved women with diabetes in all SD items and the rate of involvement were higher among type 1 diabetic cases [7]. Further research in the U.S has indicated that women with type 2 diabetes may experience similar sexual functioning to women without the disease; while women with type 1 diabetes may report more SD including dyspareunia [26]. Studies on men with diabetes also have indicated high occurrence of SD in the patients. Selvin reported that the prevalence of erectile dysfunction was over 50% in men with diabetes in the U.S [27]. A study from the Netherlands stated that the prevalence of erectile dysfunction in patients with type 2 diabetes was about 41.3% [28]. Studies from Saudi diabetic patients reported moderate to severe erectile dysfunction among 80 to 90% of the patients [29,30]. However, the prevalence of erectile dysfunction among diabetic men varies between 35-90% [19]. In addition, in this study retrograde ejaculation was reported in 15 diabetic male cases that indicate a normal ejaculation. In general, compared to most previous research this study found a higher prevalence of SD among men and women with diabetes (88% of women and 77% of men). There is a limited data regarding SD prevalence in patients with diabetes of both genders in Iran. The high prevalence could be explained by the fact that we did not control for psychological factors affecting SD in diabetes. Moreover this study investigated a small and heterogeneous sample. Further research should focus on all influential factors that might relate to prevalence of SD in diabetic patients in Iran.

In this study we did not find any significant statistical relationship between age and SD. However higher age groups experienced elevated rates of SD (Table 2). Other research has reported age as a prognostic factor on prevalence of SD among patients with diabetes. El-Sakka in his study reported that 32% of diabetic male patients less than 50 years had erectile dysfunction compared to 67.6% incidence in patients over 50 years [31]. The effect of increased age on prevalence of SD in patients with diabetes of both genders are well documented [25,32-34].

Moreover, no statistical associations were found between clinical characteristics of patients including diabetes status, duration of diabetes and hypertension with SD. Similarly a study from Turkey indicated that no risk factor predicted SD in diabetic women [7]. Enzlin from Belgium has reported also that SD in type 1 diabetic women did not correlate with age, BMI, duration of diabetes, glycemic control, using medication, menopausal status or complications [8]. Nonetheless, it has been suggested that a normal glycemic control in type 2 diabetic women would be fundamental to restoring normal sexual activity in diabetic women [5]. Besides, in men with diabetes there have been significant associations between control of diabetes mellitus and normal total testosterone levels at 3 and 6 months follow-up visits [35]. Studies have shown that poor glycemic control, longer duration of diabetes, and chronic diabetic complications such as hypertension are related to elevated incidence of SD in female and male diabetic cases [25,30,35-38].

The findings of this study showed that type of diabetes had a significant effect on SD and all the type 1 diabetic cases presented with SD (Table 2). This is similar to findings by other researchers indicating a higher incidence of SD among type 1 diabetic patients [7,26,39].

Considering the high prevalence of SD among patients with diabetes, it seems the management of these disorders should be acknowledged more precisely in health care setting. It is argued that in the absence of definitive treatment evidence, psychological counseling along with hormonal therapies may relieve the SD in female patients with diabetes [40,41]. In general patients with diabetes may benefit from educational interventions to reduce the SD impact on their personal life [42]. In addition, cognitive behavior therapy, problem solving skills and improving family communications might help to minimize the outcomes of SD among the patients. Indeed effective interactions with diabetic patients who suffer from sexual problems remain as the main task of health care workforce.

This study provided useful data on SD using standard instruments in a group of patients with diabetes of both genders. As discussed in this study no major differences were found between SD and age, diabetes status, duration of diabetes and hypertension. In addition, glycemic control did not show a significant association with SD in both genders. Such observations might be explained by the fact that the small sample size and heterogeneity of the sample did not allow to detect statistical significance between SD and age, diabetes status, duration of diabetes and hypertension. Furthermore evaluating SD among male cases was somehow restricted where one item (intercourse satisfaction) was not included in the questionnaire. Defining GH level less than 10% in the study sample also would be considered as another explanation that did not reveal significant association between SD and diabetes status. In addition we did not control for the presence of depressive symptoms and some risk factors (such as smoking) for developing SD in diabetic patients. Moreover this study assessed patients with type 1 and 2 diabetes of both genders. As most patients in this study were detected with type 2 diabetes, we could not find any significant associations between SD and demographic and clinical variables. However all patients with type 1 diabetes presented with SD and it was found that there is a significant association between type of diabetes and SD. Perhaps future studies should include all risk factors so that one gets more precise findings. Finally, to have a better insight into the association between SD and variables studied, a multivariate analysis is recommended in order to exclude the effects of covariates on the association of variables. Therefore future studies on the topic should overcome these limitations and planning prospective studies would be of great importance and worthiness. Above all one should be aware that in this study sexual dysfunctions in females were not diagnosed according to the DSM-IV and AFUD diagnostic criteria, using a non-standard cut-off score in the FSFI scale (where the standard cut-off score should be less than 26.55) and not studying the presence of distress in the subjects. In some instances the scores in the FSFI do not correlate with the true presence of SD. In fact using different instruments for assessing sexual dysfunctions can produce different estimates [43].

## Conclusion

The findings of this study showed that SD prevalence was high in diabetic patients of both genders and the glycemic control did not correlate with the frequency of SD in the study population. It is recommended that SD should be addressed more precisely in health care practice in Iran.

## Competing interests

The authors declare that they have no competing interests.

## Authors' contributions

MZR carried out the study, and involved in drafting the manuscript. MV contributed to the statistical analysis and writing the manuscript. AM contributed to the analysis, edited the paper and provided the final version. All authors read and approved the final manuscript.
